# eDNA-qPCR Reveals Spatial Biomass and Habitat Associations of the Endangered *Brachymystax lenok tsinlingensis* in Zhouzhi Heihe River

**DOI:** 10.3390/ani16131957

**Published:** 2026-06-24

**Authors:** Hu Zhao, Xiaoran An, Kunyang Zhang, Han Zhang, Jie Deng, Jianlu Zhang, Cheng Fang, Fei Kong, Wei Jiang, Qijun Wang, Xin Ding, Hongying Ma

**Affiliations:** 1 Shaanxi Key Laboratory of Qinling Ecological Security, Shaanxi Institute of Zoology, Xi’an 710032, China; zhaohu2007@126.com (H.Z.); hanhanr9@163.com (H.Z.); dengjie0311@xab.ac.cn (J.D.); zhangjianlu@xab.ac.cn (J.Z.); f-chin@163.com (C.F.); k.coffee@163.com (F.K.); jiangwei197981@163.com (W.J.); wqjab1@126.com (Q.W.); 2 Fisheries Research & Technology Extension Center of Shaanxi, Xi’an 710086, China; ax121121@163.com; 3 College of Animal Science and Technology, Northwest A&F University, Yangling 712100, China; xoozhan@nwafu.edu.cn; 4 Xi’an Zhouzhi Aquatic Products Station, Zhouzhi 710499, China; www3459@126.com

**Keywords:** *Brachymystax lenok tsinlingensis*, eDNA-qPCR, biomass distribution, habitat correlates, water depth, conservation

## Abstract

*Brachymystax lenok tsinlingensis* is an endangered salmonid endemic to China. Traditional trapping methods frequently fail to detect this rare fish in low-density mountain streams, hampering evidence-based conservation. Here, we employed environmental DNA quantitative PCR (eDNA-qPCR) with species-specific primers to assess the spatial biomass distribution of this species in the Zhouzhi Heihe River. Concurrently, we surveyed plankton, benthic macroinvertebrates, and physicochemical water parameters. eDNA detected the target species at 12 of 14 sites, with reliable quantification achieved at 9 sites, suggesting that the method may be more effective than conventional trapping for detecting this species under the studied low-density conditions. eDNA-derived relative biomass exhibited pronounced spatial heterogeneity, ranging from 6.0 × 10^−4^ to 1.5 × 10^−2^ g/cm^3^. Water depth showed a significant positive association with biomass (r = 0.5347), whereas phytoplankton Shannon diversity (a measure of species richness and evenness) was significantly negatively correlated (r = −0.5447). Flow velocity displayed a negative trend that did not reach statistical significance (r = −0.5009). Plankton and benthic communities indicated overall ecological conditions but did not directly explain the observed spatial variation in fish biomass. These findings indicate that the spatial pattern of *B. lenok tsinlingensis* is primarily shaped by local physical habitat structure, with deeper, hydraulically more complex channel units serving as key microhabitats. eDNA-qPCR thus represents an effective, low-disturbance monitoring tool for this endangered cold-water fish and provides a scientific basis for targeted habitat protection and restoration.

## Simple Summary

The Qinling lenok is an endangered fish living in cold mountain rivers in China. Traditional trapping often fails to detect this rare fish in low-density areas. In this study, we used environmental DNA (eDNA)—a method that detects DNA traces left by fish in water—to survey the Zhouzhi Heihe River. We found that eDNA detected the fish at most sampling sites, while traps caught it at only a few places, suggesting higher detection sensitivity under low-density conditions, though true population abundance remains unknown. Deeper water was linked to more eDNA (indicating more fish), whereas faster flow and higher algal diversity were linked to less eDNA. Not all sites yielded consistent results, indicating that local habitat conditions influence the method. Our findings suggest that deeper stream areas are priority habitats for conservation. eDNA is an effective, low-disturbance tool for long-term monitoring of this endangered species.

## 1. Introduction

*Brachymystax lenok tsinlingensis* (hereafter *B. lenok tsinlingensis*) is a salmonid fish endemic to China and belongs to the genus *Brachymystax*. Its distribution is primarily confined to the tributary systems of the Wei River on the northern slopes and the Han River on the southern slopes of the Qinling Mountains. This range extends to include tributaries in Zhangjiachuan, Minxian, and Gangu counties within the upper Wei River basin of Gansu Province [1]. The evolutionary origin of this species dates to the Quaternary glacial period (~2.5 million years ago). Ancestral salmonids migrated from cold Northern Hemisphere marine environments into the Qinling region to spawn. Climatic and geological events subsequently isolated these populations in mountain streams, where adaptation and speciation produced the distinct species now recognized as *B. lenok tsinlingensis* [2,3]. Intensifying anthropogenic pressures, including habitat encroachment and river fragmentation associated with socio-economic development, have severely threatened this species. As a Chinese endemic and one of the world’s southernmost salmonids, together with the Sichuan taimen (*Hucho bleekeri*), *B. lenok tsinlingensis* has undergone severe population fragmentation, leading to reduced genetic diversity and a significantly heightened risk of extinction [4,5,6,7]. In recognition of its precarious status, *B. lenok tsinlingensis* was designated as a Class II National Key Protected Wild Animal in China in 1988. Its conservation priority was reaffirmed by its inclusion in the updated List of National Key Protected Wild Animals (2021 Edition), underscoring the species’ continued endangered status in the wild [8].

In recent years, both passive and active measures have been adopted to conserve *B. lenok tsinlingensis*, including the establishment of nature reserves, artificial propagation, and population restoration efforts. Passive conservation primarily involves establishing nature reserves with the species as a primary conservation target and strictly protecting its core habitats [5], whereas active conservation includes artificial propagation, broodstock rearing, and genetic background assessment [9,10]. However, these efforts remain constrained by the lack of continuous, low-disturbance methods for monitoring population trends in the wild, spatial distribution, and changes in key habitats. In addition, climate change may further restrict the species’ potentially suitable habitat to upstream reaches and high-elevation tributaries, making high-altitude cold-water streams increasingly important for its long-term persistence [11].

Current research on *B. lenok tsinlingensis* spans multiple fields, including morphological analysis [12], habitat and life-history ecology [5,13], physiological responses to thermal stress [14,15], artificial propagation and aquaculture techniques [10,16], conservation biology [17], and molecular biology [18]. In terms of habitat ecology, field investigations have shown that adults are more commonly found in montane streams at higher elevations with steeper gradients, greater canopy cover, coarser substrates, and more developed step-pool units, and that their density is positively correlated with slope, step-pool density, elevation, substrate roughness, and canopy cover [5]. Studies on larval fish have further shown that their abundance is significantly higher in tributaries than in the main stem and is significantly negatively correlated with flow velocity [13]. Additionally, *B. lenok tsinlingensis* is highly sensitive to temperature variation; even short-term winter warming can significantly suppress its response to chemical alarm cues [7], while climatic factors are also considered key constraints on the species’ large-scale suitable habitat distribution [11]. Meanwhile, local environmental variables such as water temperature, flow velocity, and water depth constitute important ecological determinants of habitat characteristics in montane stream fishes [19]. Collectively, these studies have provided an important foundation for understanding the habitat requirements of *B. lenok tsinlingensis*; however, continuous, low-disturbance monitoring of spatial population distribution and its key drivers in natural rivers remains limited, largely because most existing studies still rely on traditional sampling methods and focus primarily on adults or a single life-history stage.

Environmental DNA (eDNA) detection technology has advanced substantially, with primary applications in monitoring population distribution [20,21], estimating species abundance and biomass [22,23], and tracking life-history processes [24,25]. eDNA has been demonstrated to serve not only for assessing fish biomass in controlled aquarium experiments [26,27], but also for detecting and quantifying target species in both lentic and lotic environments, including lakes [28,29] and rivers [30,31]. Compared with traditional netting, electrofishing, or direct observation, eDNA-qPCR offers several advantages, including non-invasiveness, high sensitivity, and suitability for monitoring low-density or cryptic species [32]. However, in lotic ecosystems, the spatial interpretation of eDNA signals may still be influenced by downstream transport and related hydrodynamic processes, thereby increasing the difficulty of inferring local occurrence patterns [33]. Previous research has indicated that fish eDNA can be transported downstream to some extent in flowing waters; therefore, detected eDNA signals do not necessarily correspond exactly to the immediate, local presence of the target species at a given sampling site [34]. Despite the broad application of eDNA technology in aquatic biomonitoring, studies focusing on *B. lenok tsinlingensis* remain very limited. Our research group has not only conducted preliminary work on eDNA quantification for this species under aquaculture conditions but has also carried out initial field validation in the Heihe River of Zhouzhi, establishing a Ct-based technical workflow for estimating the distribution and biomass of *B. lenok tsinlingensis* [35]. Nevertheless, systematic studies are still lacking on how eDNA-qPCR performs in natural river systems in terms of both sensitive detection and reliable quantification, and on how eDNA-derived biomass estimates relate to local habitat conditions and hydrodynamic processes.

The Zhouzhi Heihe River is the largest tributary of the Wei River within Zhouzhi County, Xi’an. Its basin spans an elevation from 600 to over 3500 m and is predominantly covered by dense forests, with multiple sections designated as national nature reserves. Characterized by clear water and relatively low levels of pollution, the river provides ideal habitat for rare fish species such as *B. lenok tsinlingensis* [36]. In its lower reaches, the Heihe Reservoir serves as a critical source of drinking water for Xi’an and irrigation water for surrounding agricultural areas [37]. Plankton and benthic macroinvertebrate communities are key indicators of water quality and river health, with variation in their composition reflecting trophic status and pollution levels in aquatic systems. In particular, benthic macroinvertebrate community structure is closely linked to local environmental conditions and has been widely used as an indicator of water quality in rivers [38]. Meanwhile, zooplankton distribution is strongly influenced by dissolved oxygen, salinity, nutrients, water depth, and turbidity, and can therefore be used in the biological assessment of water quality [39]. Previous studies have shown that fish, macroinvertebrates, and benthic algal communities all respond to riverine environmental gradients and trophic conditions. Fish communities are closely associated with hydromorphological and nutrient variables [40]. Meanwhile, benthic algae, macroinvertebrates, and fish can together serve as indicator groups of changes in river ecological status [41]. Therefore, integrating plankton and benthic macroinvertebrate community data with local environmental factors and eDNA-qPCR may provide a more comprehensive understanding of species distribution, habitat conditions, and broader ecological background in montane streams, particularly with respect to water quality, hydrodynamic setting, and fish occurrence.

Based on this background, the present study combined eDNA-qPCR, conventional trap surveys, physicochemical measurements, and surveys of plankton and benthic macroinvertebrate communities in the Zhouzhi Heihe River to address four objectives: (1) to compare the detection performance of eDNA-qPCR with that of conventional trapping for *B. lenok tsinlingensis*; (2) to examine how local physical habitat and hydrodynamic variables, especially water depth and flow velocity, are associated with eDNA-derived biomass; (3) to assess whether plankton and benthic assemblages function primarily as indicators of ecological condition and water-quality background rather than as direct predictors of fish biomass; and (4) to evaluate whether reliable eDNA quantification varies among river habitats under LOD/LOQ constraints. Accordingly, we hypothesized that (H1) eDNA-qPCR would provide more sensitive detection than conventional trapping for this low-density endangered fish; (H2) local physical habitat, particularly deeper water and lower-flow reaches, would be associated with higher eDNA-derived biomass; (H3) plankton and benthic assemblages would mainly indicate ecological background and water-quality conditions, and their diversity indices would not necessarily correspond directly to spatial variation in fish biomass; and (H4) intense hydrological disturbance and high-flow habitats would reduce the reliability of quantitative eDNA detection in mountain rivers. This framework allows the present study to evaluate the species from three connected perspectives: detection sensitivity, habitat association, and the environmental constraints on quantitative eDNA performance.

## 2. Materials and Methods

### 2.1. Water Sampling Sites

**Figure 1:**
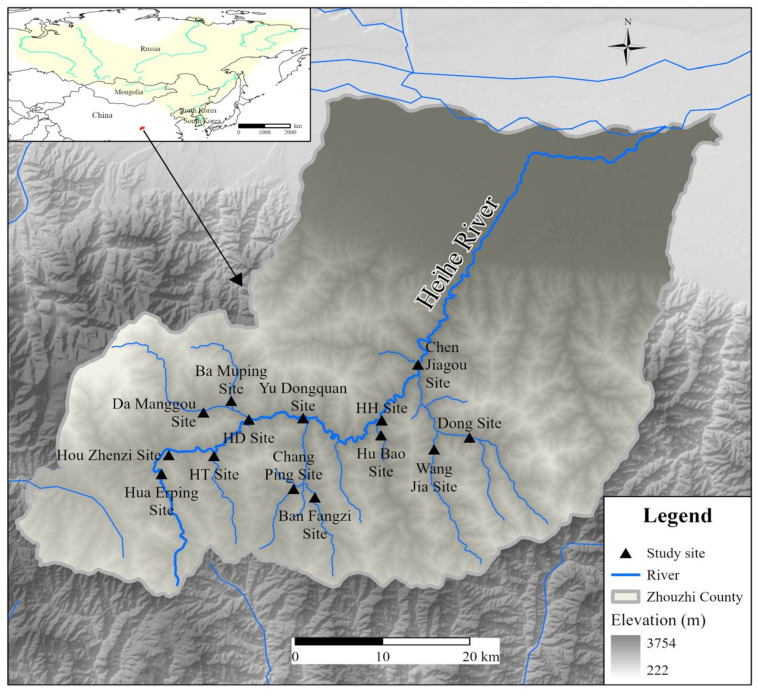
Collection sites for *B. lenok tsinlingensis* eDNA in the Zhouzhi Heihe River. (The map in the top-left corner shows that the red area within China represents the habitat of *B. lenok tsinlingensis*. This figure was generated using ArcGIS (10.8).

In June 2025, triplicate surface water samples (2 L each) were collected from fourteen locations along the Heihe River in Zhouzhi County and were immediately filtered on-site through a 0.45 µm membrane filter (Thermo Scientific, 145-2045, Waltham, MA, USA) using a centrifugal filter unit (Newking, DR-B80301, Fuzhou, China). At each sampling site, 2 L of distilled water was separately filtered through the same procedure to serve as a negative control. The selection of sampling sites was strictly based on the habitat preferences of *B. lenok tsinlingensis*: (1) Elevation filtering—all sites were located in mid- to high-elevation (above 600 m above sea level) cold areas to meet the species’ requirement for low-temperature waters; (2) Habitat type—sites were concentrated in the upper tributaries and headwater areas of the Heihe River to match the species’ preference for gravel substrates and step-pool environments; and (3) Ecological integrity—the selected sites facilitated simultaneous surveys of water physicochemical parameters, plankton, and benthic macroinvertebrates. The locations of the sampling sites are summarized in Table 1, and their spatial distribution across the main channels of the Zhouzhi Heihe River is illustrated in Figure 1. Following filtration, the membrane filters were immersed in 95% ethanol, transported to the laboratory under low-temperature conditions, and promptly stored at −20 °C for subsequent analysis. All filtration equipment was carefully rinsed with distilled water between filtration operations to prevent cross-contamination.

### 2.2. eDNA Extraction and Quantification

Environmental DNA (eDNA) was extracted using a DNeasy Blood and Tissue Kit (Qiagen, Hilden, Germany). Filters were removed from ethanol, air-dried for 6–7 h, and aseptically cut in half. Each half was placed in a 2 mL microcentrifuge tube with 500 µL ATL buffer and minced. Digestion was performed with 30 µL Proteinase K at 56 °C for 48 h. Subsequent steps followed the manufacturer’s protocol, with the following modification: after digestion, 500 µL of AL buffer and 500 µL of absolute ethanol were added to each lysate. Extraction negative controls (filter-free) were processed alongside the samples to monitor for contamination. DNA concentration was determined using a Nanodrop 2000 spectrophotometer (Thermo Fisher Scientific, Waltham, MA, USA).

Quantitative real-time PCR (qPCR) was performed using the Premix Ex Taq (Probe qPCR; TaKaRa Bio, Dalian, China) on a Real-Time PCR system. To prevent contamination, sample processing (pre-amplification), PCR setup, and post-amplification analysis were conducted in separate, dedicated rooms. Each 25 µL reaction contained: 12.5 µL of Premix Ex Taq, 8.5 µL of ddH_2_O, 10 nM TaqMan probe, and 5 nM of each primer (primer sequences were derived from our previous study [35]. The thermal cycling protocol involved an initial denaturation at 95 °C for 30 s, followed by 40 cycles of denaturation at 95 °C for 30 s, annealing at 60 °C for 30 s, and extension at 72 °C for 30 s. All samples were run in four to eight technical replicates, and the mean quantification cycle (Ct) value was used for analysis. An extraction negative control and a no-template control were included with each batch and on every qPCR plate, respectively. A sample was regarded as negative under these conditions if no exponential amplification was observed within 40 cycles.

Following the recommendations of Klymus et al. for eDNA qPCR assays, we defined the limit of detection (LOD) as the lowest target DNA concentration that can be reliably detected with a 95% detection probability, and the limit of quantification (LOQ) as the lowest target DNA concentration that can be reliably quantified under the stated experimental conditions while meeting a predefined precision threshold; in this study, acceptable precision was defined as a coefficient of variation (CV) < 35%. Using the discrete-threshold approach, the LOD and LOQ were taken as the lowest standard concentrations yielding ≥95% positive technical replicates and meeting the above precision criterion, respectively, with the additional constraint that the LOQ could not be lower than the LOD. The *CV* was calculated as [42]:

(1) $$CV_{\ln}=\sqrt{{\left(1+E\right)}^{{\left(SD\right)}^{2}*\ln(1+E)}-1}$$

where *E* is the qPCR efficiency, and *SD* is the standard deviation of Ct values among technical replicates.

### 2.3. Methodology for Aquatic Organism Surveys

Aquatic organism surveys were conducted to assess fish resources, plankton communities (phytoplankton and zooplankton), benthic macroinvertebrates, and fish habitats. To minimize potential cross-contamination between molecular and traditional ecological methods, all traditional biological sampling (fish, plankton, and benthos) was performed after the completion of environmental DNA (eDNA) water sample collection and filtration at each site.

#### 2.3.1. Fish Sampling and Processing

Fish were sampled using two baited traps (dimensions: 4.8 m in length, 0.45 m in width, 0.33 m in height), positioned at least 5 m apart at each sampling point. Traps were deployed overnight and retrieved the following day. Captured fish were immediately transferred to a container with MS-222 anesthetic (100 mg/L) for approximately 5 min. Following sedation, total length, standard length, and body weight were recorded using digital calipers and an electronic balance. All fish were revived in fresh river water and released at the point of capture immediately after measurement. The fish capture data were obtained from conservation monitoring conducted by the Zhouzhi Fisheries Station in the Heihe River.

#### 2.3.2. Plankton Sampling and Laboratory Analysis

##### Phytoplankton

Phytoplankton samples were collected at a depth of 0.5 m using a water sampler, with 1000 mL collected per sample. Each sample was fixed with 15 mL of Lugol’s iodine solution, transferred to a labeled plastic bottle, and stored in the dark at room temperature for transport. In the laboratory, samples were settled for more than 24 h and concentrated to 50 mL. For analysis, a 0.1 mL subsample was examined under an Olympus BX43 microscope equipped with a phytoplankton counting chamber for taxonomic identification and cell density enumeration.

##### Zooplankton

Zooplankton were collected by towing a 64-μm mesh plankton net at a depth of 0.5 m, filtering approximately 20 L of water. The concentrated sample was preserved with 2 mL of formaldehyde in a 100 mL plastic bottle and stored in the dark at room temperature. For analysis, a 1 mL subsample was taken from the concentrate and examined under an Olympus BX43 microscope fitted with a counting chamber for taxonomic identification and enumeration.

#### 2.3.3. Benthic Macroinvertebrate Sampling

Benthic macroinvertebrates were collected from shallow, rocky habitats using a Surber sampler (No. 60 mesh, 250 μm; frame size 30 × 30 cm). A total area of 1 m^2^ was surveyed at each site. Samples were initially sieved on-site, transferred to white sorting trays for preliminary examination, preserved in 99% ethanol, and stored in 50 mL specimen bottles. In the laboratory, organisms were identified to the lowest feasible taxonomic level (typically species or genus) using a combination of a stereomicroscope and an Olympus BX43 compound microscope. Wet biomass was measured using a precision analytical balance (0.0001 g accuracy), and both abundance (ind./m^2^) and biomass (g/m^3^) were subsequently calculated.

#### 2.3.4. Taxonomic References

Taxonomic identification was performed using standard morphological keys and authoritative regional monographs. Phytoplankton were identified primarily to the genus level with reference to *Systematics*, *ArcGISlassification and Ecology of Freshwater Algae in China* [43]. Zooplankton (including rotifers, cladocerans, and copepods) were identified to the lowest possible taxonomic level (typically species or genus) based on *Freshwater Rotifera of China* [44], *Fauna Sinica: Freshwater Cladocera* [45], *Fauna Sinica: Freshwater Copepoda* [46], and supplementary guidance from *New Techniques in Microorganism Monitoring* [47]. Benthic macroinvertebrates were identified using *Studies on Chinese Naididae* [48] and the comprehensive text *Aquatic Entomology* [49]. Although some references are classic works, they remain the standard taxonomic authorities for the freshwater fauna of the study region.

#### 2.3.5. Water Quality Measurements

At all 14 sampling sites, water quality parameters, including temperature, pH, turbidity, dissolved oxygen, and flow velocity, were measured in situ using a portable HORIBA U-52 multiparameter water quality meter (Horiba, Ltd., Kyoto, Japan). In addition, water depth was recorded at the thalweg using a Dingfeng leveling staff (Model 0708, 3 m length, Hangzhou, China). All measurements were performed in triplicate, with the mean value of each parameter being used for subsequent analysis.

### 2.4. Data Processing and Analysis Methods

Biomass was estimated by inputting the raw qPCR Ct values into a pre-established calibration curve published in our prior study [35]:

(2) $$y=2\times 10^{11}\cdot e^{-1.102x}$$

where *y* denotes biomass (g/cm^3^) and *x* represents the Ct value.

This equation, derived from controlled laboratory experiments, allows for the direct translation of Ct values into quantitative biomass estimates. All calculations were performed in MATLAB R2018b, and the results were compiled to generate a biomass value for each sampling site.

To quantify biodiversity, the Shannon-Wiener index (*H′*) [50], Margalef richness index (*D*) [51], and Pielou evenness index (*J*) [52] were applied to the community data of plankton and benthic macroinvertebrates. The corresponding formulas are given below

(3) $$H^{\prime }=-\sum_{i=1}^{S}P_{i}\cdot \ln P_{i}$$

(4) $$D=\left(S-1\right)/\log_{2}N$$

(5) $$J=H^{\prime }/\log_{2}N$$

where *P_i_* is the proportion of individuals of the *i-th* species in the sample, *S* denotes the total number of species, and *N* represents the total number of individuals.

After compiling a raw data matrix of all environmental variables and fish biomass in Excel (Microsoft, Redmond, WA, USA), we performed Pearson’s product–moment correlation analysis between these variables and the biomass of *B. lenok tsinlingensis* using MATLAB R2022a (The MathWorks, Natick, MA, USA). The significance of each correlation coefficient was assessed using a two-tailed *t*-test with n−2 degrees of freedom. A *p*-value ≤ 0.05 was considered statistically significant.

## 3. Results

### 3.1. Biomass of B. lenok tsinlingensis Using qPCR Assay

To ensure the reliability of the quantitative estimates, predefined detection limits were applied. Based on prior assay validation, theLOD was set at $1.1\times 10^{2}$ copies/μL, and the LOQ at $1.1\times 10^{3}$ copies/μL. Using these criteria, eDNA of *B. lenok tsinlingensis* was detected at 12 of the 14 sampling sites. Reliable quantification (≥LOQ) was achieved at nine sites: Hua Erping, Hou Zhenzi, HD, Yu Dongquan, Ban Fangzi, HT, Chang Ping, Chen Jiagou, and HH. Concentrations below the LOQ but above the LOD were observed at three sites (Da Manggou, Ba Muping, and Hu Bao), indicating detectable but not reliably quantifiable levels. No eDNA was detected at the Dong site (concentration: 68 copies/μL, below the LOD) or at the Wang Jia site (no amplification) (Table 2). Only sites with eDNA concentrations above the LOQ were included in the reliable biomass quantification used for subsequent environmental correlation analysis.

In a complementary trap survey, *B. lenok tsinlingensis* was physically captured at only two locations: one individual in the Ban Fangzi site (20.2 cm total length, 54.2 g body weight) and one in the Chang Ping site (5.1 cm total length, 1.3 g body weight).

**Figure 2:**
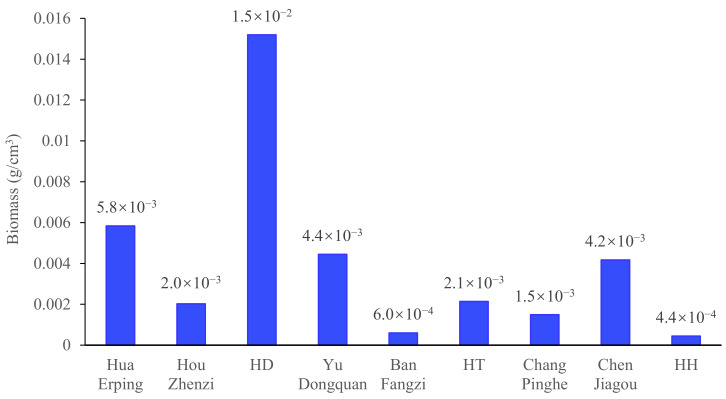
Biomass of *B. lenok tsinlingensis* in the Zhouzhi Heihe River. (HT, the confluence of the Heihe River and the Taiping River; HD, the confluence of the Heihe River and the Da Manggou River; HH, the confluence of the Heihe River and the Hubao River.).

This study estimated the biomass density of *B. lenok tsinlingensis* in the Zhouzhi Heihe River using an environmental DNA (eDNA)-based quantitative PCR (qPCR) approach. Ct values obtained from 9 sampling sites were converted to biomass density via a pre-established regression model (*y* = 2 × 10^11^·e^−1^·^102*x*^; R^2^ = 0.9987) [35]. Biomass density varied across river sections, ranging from 1.5 × 10^−2^ g/cm^2^ at the HD site to undetectable levels at the Wang Jia site. The highest value occurred at the HD site (the confluence of the Heihe River and the Da Manggou River, 1.5 × 10^−2^ g/cm^3^), followed by Hua Erping site (5.8 × 10^−3^ g/cm^3^), Yu Dongquan site (4.4 × 10^−3^ g/cm^3^), and Chen Jiagou site (4.2 × 10^−3^ g/cm^3^). Intermediate levels were observed in the HT site (the confluence of the Heihe River and the Taiping River, 2.1 × 10^−3^ g/cm^3^), Hou Zhenzi site (2.0 × 10^−3^ g/cm^3^), and Chang Ping site (1.5 × 10^−3^ g/cm^3^). Lower values were recorded in Ban Fangzi site (6.0 × 10^−4^ g/cm^3^), HH site (the confluence of the Heihe River and the Hubao River, 4.4 × 10^−4^ g/cm^3^) (Figure 2).

### 3.2. Results of the Survey

#### 3.2.1. Phytoplankton Indicators

Based on water samples collected in July 2025 from 14 sampling sites in the Zhouzhi Heihe River Basin, a total of 26 phytoplankton species were identified and assigned to five phyla: Bacillariophyta, Cyanophyta, Chlorophyta, Cryptophyta, and Dinophyta (Table S1). Bacillariophyta was the most species-rich group (16 species; 61.54% of the total), followed by Cyanophyta (4 species; 15.38%), Chlorophyta (3 species; 11.54%), Cryptophyta (2 species; 7.69%), and Dinophyta (1 species; 3.85%).

Phytoplankton community composition varied significantly among sites. Species richness ranged from 1 to 16 taxa. Bacillariophyta dominated both cell density and biomass at most sites (e.g., Da Manggou and Chang Ping site). Notably, only diatoms were detected at Hua Erping site, HT site, Ban Fangzi site, and Dong site, indicating highly simplified community structures. In contrast, other groups showed marked site-specific enrichment. Cyanophyta became the absolutely dominant group in cell density at Hou Zhenzi site (1.8 × 10^5^ cells/L) and Chen Jiagou site (7.5 × 10^4^ cells/L) (Table 3). Hou Zhenzi also recorded highest cyanobacterial biomass (1.0 × 10^3^ mg/L) among all sites (Table 4). Additionally, Chlorophyta and Cryptophyta were detected at Ba Muping site, while Chlorophyta was also present at both the Wang Jia and Yu Dongquan sites.

**Figure 3:**
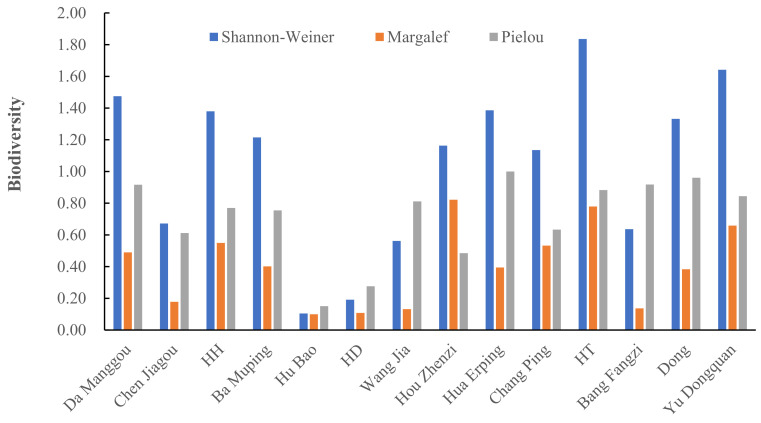
Phytoplankton diversity indices at different sites in the Zhouzhi Heihe River.

Diversity indices showed substantial spatial variation among sites. The Shannon–Wiener index (H′) ranged from 0.10 to 1.35 across the basin. Da Manggou site, Hua Erping site, and Yu Dongquan site exhibited relatively high diversity (H′ > 1.3) and evenness (J > 0.84), whereas H′ values were extremely low at Hu Bao site and HD site. The Margalef richness index (D) varied considerably, with the lowest value at Hu Bao (D = 0.0996) and the highest at Hou Zhenzi (D = 0.8220). However, Hou Zhenzi site exhibited relatively low evenness (J = 0.4849), indicating dominance by a few highly abundant species (Figure 3). Across all sites, the mean values of H′, D, and J were 1.0522, 0.4047, and 0.7155, respectively.

Overall, the phytoplankton communities can be classified into several distinct structural types indicating different aquatic environmental conditions. Sites such as Da Manggou site, Hua Erping site, and Yu Dongquan site, characterized by high diversity and evenness with minimal cyanobacterial occurrence, indicate clean and relatively stable waters. Hou Zhenzi site displayed typical eutrophic features, with extremely high cyanobacterial and dinoflagellate biomass, the highest richness, but low evenness. Communities at Hu Bao site and the HD site were highly simplified, while Chen Jiagou site was mainly characterized by markedly elevated cyanobacterial cell density. The remaining sites exhibited transitional community patterns, characterized by the coexistence of multiple phyla and intermediate levels of diversity. Collectively, the pronounced spatial differentiation of phytoplankton community structure indicates substantial variability in trophic status and environmental quality across the basin, with clean and eutrophic conditions coexisting.

#### 3.2.2. Zooplankton Indicators

The zooplankton composition across the 14 sampling sites in the Heihe River, Zhouzhi, was species-poor, with only 8 species identified from 3 phyla (Table S2). The overall mean density was extremely low (1.08 ind./L). Cladocerans were completely absent at all sites, while rotifers and copepods occurred at very low mean densities of 0.57 ind./L and 0.22 ind./L, respectively. Their distribution was spatially patchy and sporadic, with detectable abundances recorded only at a few locations (e.g., rotifers: 2 ind./L at Hua Erping site and 3 ind./L at Da Manggou site; copepods: 1 ind./L at Hou Zhenzi site). Biomass followed a similar pattern, averaging only 1.2 × 10^−3^ mg/L overall. Mean biomass values of rotifers and copepods were 1.1 × 10^−3^ mg/L and 0.9 × 10^−3^ mg/L, respectively, with elevated values likewise restricted to isolated sites (e.g., rotifer biomass: 2.4 × 10^−3^ mg/L at both Hua Erping and Hu Bao sites; copepod biomass: 3.0 × 10^−3^ mg/L at Hou Zhenzi site, Ban Fangzi site, and Chen Jiagou site). Across the vast majority of sites, densities and biomass of all zooplankton groups were at or near the detection limit. Overall, this depauperate, low-abundance zooplankton assemblage indicates a markedly simplified aquatic food web.

#### 3.2.3. Macrobenthos Indicators

A total of 26 benthic species were identified across 14 sampling sites in the Heihe River Basin of Zhouzhi County. The macroinvertebrate community was structurally intact and taxonomically diverse, being dominated by pollution-sensitive aquatic insects. Ephemeroptera (mayflies) emerged as the most abundant group, accounting for approximately 63% of total abundance and represented by 10 genera. Pollution-sensitive indicator taxa such as Ephemeroptera and Trichoptera were prevalent, whereas pollution-tolerant groups like Diptera and Coleoptera did not dominate—a pattern further supported by biomass data (Table S3). This community composition suggests well-oxygenated water, low organic pollution, and generally good water quality.

**Figure 4:**
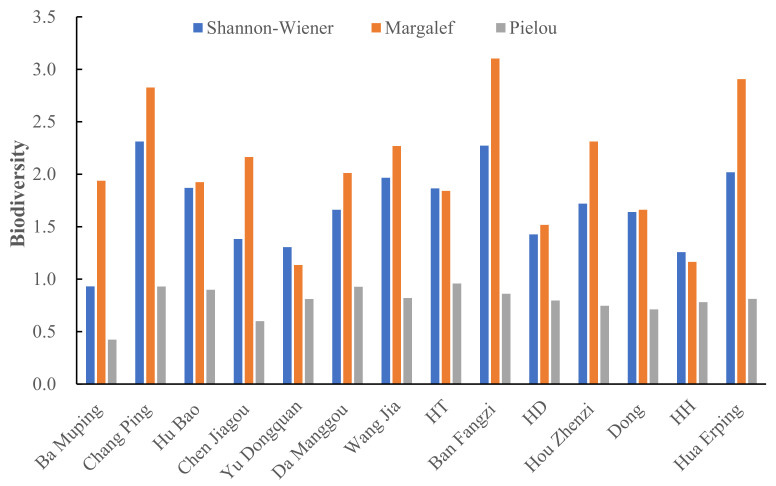
Macrobenthos biodiversity indicator diagram of the Zhouzhi Heihe River.

Biodiversity indices revealed pronounced spatial heterogeneity among the sampling sites. The Shannon–Wiener index (H′) varied from 0.93 at Ba Muping site to 2.31 at Chang Ping site, with Ban Fangzi site also exhibiting high diversity (H′ = 2.27). The Margalef richness index (D) was highest at Ban Fangzi site (3.10) and remained high at Hua Erping site (2.91) and Chang Ping site (2.83), whereas a relatively low value occurred at the HH site (1.16) and Yu Dongquan site (1.13). The Pielou evenness index (J) was generally elevated (>0.71) across most sites, approaching the theoretical maximum at HT site (0.96) and Chang Ping site (0.93). In contrast, Ba Muping site exhibited notably low evenness (0.42), implying possible dominance by a limited number of species and potential environmental stress (Figure 4).

Overall, benthic community composition and biodiversity indices suggest generally clean water conditions and a healthy aquatic habitat in the Heihe River.

### 3.3. Habitat Factors Influencing the Biomass of B. lenok tsinlingensis

By measuring a suite of abiotic factors (including water temperature, pH, altitude, water depth, turbidity, dissolved oxygen, and flow velocity) as well as biotic factors such as phytoplankton and macrobenthos diversity, key habitat conditions were characterized (Table 5).

As shown in Table 6, the correlation coefficients indicate that, among the various environmental factors, water depth (Dep) exhibited the strongest positive correlation with the biomass of *B. lenok tsinlingensis* (r = 0.5348, *p* ≤ 0.05), reaching statistical significance. This suggests that greater water depth is associated with higher fish biomass, indicating that deep pools serve as critical habitats for this species.

Phytoplankton diversity (H′_P_) showed a statistically significant negative correlation with biomass (r = −0.5447, *p* ≤ 0.05). Specifically, higher phytoplankton diversity corresponded to lower fish biomass, which may be indirectly related to water nutrient conditions. Flow velocity (F) demonstrated a moderate negative correlation with biomass (r = −0.5009, 0.05 < *p* ≤ 0.10), approaching statistical significance, suggesting that reaches with higher flow velocity are less suitable for this fish, which appears to prefer slow-flowing or still-water environments.

The remaining environmental factors did not reach statistical significance (*p* > 0.10), including benthic macroinvertebrate diversity (H′_BM_, r = −0.3309), turbidity (Tur, r = −0.2812), pH (r = −0.2158), water temperature (T, r = −0.1994), altitude (A, r = 0.0186), and dissolved oxygen (DO, r = −0.0429).

In summary, water depth and phytoplankton diversity are key factors significantly influencing the biomass distribution of *B. lenok tsinlingensis* in the Zhouzhi Heihe River, while flow velocity also exhibits a near-significant negative trend.

## 4. Discussion

The present study showed clear spatial heterogeneity in the eDNA-derived biomass of *B. lenok tsinlingensis* among the sampling sites in the Zhouzhi Heihe River. Among the measured environmental variables, the water depth was positively and significantly correlated with biomass. In contrast, the phytoplankton Shannon index was negatively and significantly correlated with biomass, while flow velocity showed a near-significant negative trend. These results suggest that the spatial pattern of eDNA-derived biomass for this species is primarily constrained by local physical habitat conditions, while also being influenced by broader ecological gradients and hydrodynamic processes. The positive correlation between water depth and biomass is in agreement with previous studies that demonstrate this species’ preference for cold-water mountain streams, upper tributaries, and structurally complex channel habitats, such as step-pool systems [5]. In the study reach, deeper water likely reflects not only a larger water volume but also habitat structures such as deep pools, step-pool units, and local hydraulic refugia, which provide conditions for fish to rest, take shelter, and expend lower energy [5,35]. Meanwhile, the elevated eDNA signals at confluence sites such as HD and Chen Jiagou suggest that locally suitable habitats, combined with enhanced hydrodynamic mixing at confluence zones, may jointly influence the spatial distribution of eDNA [33,53]. In this study, the higher eDNA concentration at Yu Dongquan site, a documented breeding area, was consistent with expectations and aligned with prior survey results [35]. The overall lower biomass at some sites may be primarily attributed to fish dispersal following a flood event in the basin during the previous summer, a hypothesis supported by evidence that storm-induced disturbances can reduce local population abundance [16]. One of the most notable findings of this study is the clear discrepancy between eDNA-based methods and conventional trapping, in terms of detection rate, quantification reliability, and capture results. *B. lenok tsinlingensis* eDNA was detected at 12 of the 14 sampling sites, whereas conventional trapping captured only two individuals, one at Ban Fangzi and one at Chang Ping. In addition, eDNA concentrations exceeded the LOQ at 9 sites, allowing reliable quantification; three sites (Da Manggou, Ba Muping, and Hu Bao) fell between the LOD and the LOQ, indicating detectable but not stably quantifiable signals; Dong was below the LOD, and Wang Jia showed no amplification. Together, these results suggest that eDNA-qPCR may offer higher detection sensitivity compared to trapping, although quantitative validation against independent abundance estimates is still needed, while also providing biomass information with ecological interpretive value at sites where concentrations exceed the LOQ. Importantly, these nine reliably quantifiable sites were not restricted to a single habitat type or a narrow biomass range, but covered the major spatial gradient from high to low biomass in the Heihe River, thereby supporting the subsequent identification of habitat–biomass relationships involving water depth, phytoplankton diversity, and flow velocity. Field habitat observations and supplementary site photographs further revealed that the Dong and Wang Jia reach had both experienced flood scouring and construction disturbance, leading to channel contraction and lower water levels (Table S4). Such conditions likely reduced the local eDNA input near the sampling points. By contrast, sites such as Da Manggou, Hu Bao, and Ba Muping were characterized by riffle-gravel habitats with stronger water exchange and weaker local retention, which, under potentially low local population density, are less favorable for eDNA accumulation to a stable quantification threshold. Accordingly, the coexistence of relatively high eDNA detection, very low trap-capture rates, and variable quantification reliability among sites reflects not only the different detection scales and ecological meanings of the two methods, but also suggests that intense hydrological disturbance and high-flow habitats may be important environmental constraints on stable quantitative eDNA performance in mountain rivers. For *B. lenok tsinlingensis*, these findings indicate that eDNA-qPCR is useful not only for presence/absence monitoring and hotspot identification, but also for analyzing local habitat–biomass relationships; at the same time, the quantitative correspondence between eDNA-derived biomass and true local biomass should be further calibrated with additional field data to improve interpretation under natural river conditions [54,55].

In addition to water depth, the results for phytoplankton diversity and flow velocity also carry important ecological implications. The significant negative relationship between phytoplankton Shannon diversity and biomass, together with the near-significant negative relationship between flow velocity and biomass, suggests that habitat suitability in this system is influenced not only by channel structure but also by hydrodynamic and ecological background conditions. Higher flow velocity generally implies stronger water exchange and less stable local holding conditions, which may reduce both fish residence time and the local accumulation or retention of eDNA near the sampling point, thereby contributing to lower biomass signals [13,33]. This trend is directionally consistent with previous findings that larval abundance of this species declines with increasing flow velocity in tributary habitats [13]. By contrast, the negative relationship between phytoplankton diversity and biomass is more likely to reflect variation in trophic status, water-quality background, and ecological gradients than directly available food resources. Results from the present study showed that phytoplankton communities in the Heihe River reflected a spatial background ranging from oligotrophic to more nutrient-enriched conditions, indicating that higher phytoplankton diversity is more plausibly interpreted as an indicator of environmental context than as a direct measure of resource availability for *B. lenok tsinlingensis* [56]. For salmonids in cold mountain streams, actual prey conditions are more closely related to benthic biomass, drift density, body-size composition, and seasonal emergence patterns than to phytoplankton diversity itself [57,58]. Therefore, in the present study, phytoplankton diversity is better interpreted as an indicator of broader ecological condition and water-quality variation than as a direct resource-based explanation of fish biomass.

The benthic macroinvertebrate results support the same interpretation. Although the Shannon diversity of benthic macroinvertebrates showed only a moderate negative correlation with biomass and did not reach statistical significance, the benthic assemblage as a whole still indicated that the Heihe River maintains generally good water quality and a relatively intact aquatic ecological foundation [38,59]. This finding indicates that, in the present study, plankton and benthic assemblages are more suitable as bioindicators of basin ecological status and habitat quality. In contrast, spatial variation in *B. lenok tsinlingensis* biomass is more directly associated with physical channel structure and local hydrodynamic conditions. In other words, the present study does not deny the importance of food-web support for the species, but rather that, at the scale of this site-based survey, physical habitat variables explained the spatial variation in biomass more directly than biodiversity indices.

Overall, the present results indicate that the spatial pattern of eDNA-derived biomass of *B. lenok tsinlingensis* in the Zhouzhi Heihe River is mainly associated with local physical habitat structure, with deeper and more structurally complex channel units being especially important. At the same time, the negative relationships associated with higher flow velocity and higher phytoplankton diversity further suggest that hydrodynamic conditions and broader ecological gradients jointly influence fish distribution at the reach scale as well as the spatial expression of eDNA signals. From a conservation perspective, this implies that habitat management for the species should prioritize the maintenance and restoration of deep pools, deeper water areas, and refuge habitats with strong hydraulic heterogeneity, and continue to maintain the generally higher water quality of the basin. Future studies should strengthen the correspondence between eDNA signals and true population status through repeated multi-season sampling, independent abundance surveys, and more detailed site-level habitat records, thereby enhancing the utility of eDNA-qPCR for long-term monitoring and conservation assessment of this endangered salmonid.

## 5. Conclusions

eDNA-qPCR revealed pronounced spatial heterogeneity in the biomass of *B. lenok tsinlingensis* in the Zhouzhi Heihe River, demonstrating more effective detection for this low-density endangered fish. Water depth was the clearest positive correlate of eDNA-derived biomass (r = 0.5347), while phytoplankton diversity showed a negative relationship (r = −0.5447) and flow velocity showed a negative trend that did not reach statistical significance (r = −0.5009). These results indicate that local physical habitat structure, hydrodynamic conditions, and broader ecological gradients jointly shape the spatial pattern of this species. However, not all eDNA-detected sites allowed reliable quantification, suggesting that intense hydrological disturbance and high-flow habitats may constrain quantitative eDNA performance in mountain rivers. Deeper, structurally complex channel units emerged as priority habitats for conservation. Future efforts should (1) maintain and restore deep pools and hydraulically heterogeneous refuges while preserving high water quality and (2) integrate repeated multi-season sampling with independent abundance surveys to strengthen the link between eDNA signals and true population status.

## Figures and Tables

**Table 1:** Sampling site information for eDNA analysis in the Zhouzhi Heihe River.

Number	Location	Coordinates	Altitude (m)
1	Hua Erping site	33.826553° N, 107.832346° E	1340
2	Hou Zhenzi site	33.846789° N, 107.840567° E	1288
3	HT site	33.847261° N, 107.896643° E	1170
4	HD site	33.887228° N, 107.938330° E	1077
5	Da Manggou site	33.893200° N, 107.881854° E	1265
6	Ba Muping site	33.906418° N, 107.915733° E	1202
7	Yu Dongquan site	33.890361° N, 108.005389° E	963
8	Chang Ping site	33.814817° N, 107.996285° E	1158
9	Ban Fangzi site	33.806271° N, 108.022873° E	1219
10	HH site	33.890367° N, 108.102878° E	819
11	Hu Bao site	33.874491° N, 108.102443° E	872
12	Wang Jia site	33.860821° N, 108.168671° E	995
13	Dong site	33.874596° N, 108.212104° E	981
14	Chen Jiagou site	33.950493° N, 108.145729° E	676

Note: HT site, the confluence of the Heihe River and the Taiping River; HD site, the confluence of the Heihe River and the Da Manggou River; HH site, the confluence of the Heihe River and the Hubao River.

**Table 2:** Quantification of *B. lenok tsinlingensis* eDNA using qPCR.

Number	Location	CT Value	eDNA Concentration (Copy/µL)
1	Hua Erping site	28.281	5.3 × 10^3^
2	Da Manggou site	31.386	7.9 × 10^2^
3	Hou Zhenzi site	29.243	2.9 × 10^3^
4	Ba Muping site	32.744	3.4 × 10^2^
5	HD site	27.412	9.0 × 10^3^
6	Yu Dongquan site	28.528	4.5 × 10^3^
7	Ban Fangzi site	30.349	1.5 × 10^3^
8	HT site	29.193	3.0 × 10^3^
9	Hu Bao site	31.294	8.3 × 10^2^
10	Chang Ping site	29.522	2.5 × 10^3^
11	Dong site	35.393	6.8 × 10
12	Chen Jiagou site	28.586	4.4 × 10^3^
13	HH site	30.621	1.3 × 10^3^
14	Wang Jia site	None	None

**Table 3:** Phytoplankton cell density by taxonomic group at selected sites.

Cell Density (Cells/L)	*Cyanobacteria*	*Chlorophyta*	*Bacillariophyta*	*Cryptophyta*	*Pyrrophyta*
Da Manggou site	0	0	3.5 × 10^3^	0	0
Chen Jiagou site	7.5 × 10^4^	0	0.5 × 10^3^	0	0
HH site	5.0 × 10^3^	0	4.0 × 10^3^	0	0
Ba Muping site	1.5 × 10^4^	5.0 × 10^3^	0.5 × 10^3^	0.5 × 10^3^	0
Hu Bao site	2.3 × 10^4^	0	0.5 × 10^3^	0	0
HD site	1.0 × 10^4^	0	0.5 × 10^3^	0	0
Wang Jia site	0	0.5 × 10^3^	1.5 × 10^3^	0	0
Hou Zhenzi site	1.8 × 10^5^	7.0 × 10^3^	4.5 × 10^3^	0	0.5 × 10^3^
Hua Erping site	0	0	2.0 × 10^3^	0	0
Chang Ping site	0	0	1.2 × 10^4^	0	0
HT site	0	0	8.0 × 10^3^	0	0
Ban Fangzi site	0	0	1.5 × 10^3^	0	0
Dong site	0	0	2.5 × 10^3^	0	0
Yu Dongquan site	0	0.5 × 10^3^	8.0 × 10^3^	0.5 × 10^3^	0

**Table 4:** Phytoplankton biomass by taxonomic group at selected sites.

Biomass (mg/L)	*Cyanobacteria*	*Chlorophyta*	*Bacillariophyta*	*Cryptophyta*	*Pyrrophyta*
Da Manggou site	0	0	0.9 × 10^3^	0	0
Chen Jiagou site	1.0 × 10^2^	0	0.5 × 10	0	0
HH site	0.2 × 10^2^	0	0.3 × 10^3^	0	0
Ba Muping site	0.4 × 10^2^	0.8 × 10	0.5 × 10	5 × 10^−1^	
Hu Bao site	0.8 × 10^2^	0	0.5 × 10	0	0
HD site	0.3 × 10^2^	0	0.5 × 10	0	0
Wang Jia site	0	0.2 × 10^2^	2.5 × 10^2^	0	0
Hou Zhenzi site	1.0 × 10^3^	0.1 × 10^2^	1.2 × 10^2^	0	2.0 × 10^2^
Hua Erping site	0	0	1.0 × 10	0	0
Chang Ping site	0	0	2.0 × 10^2^	0	0
HT site	0	0	1.4 × 10^2^	0	0
Ban Fangzi site	0	0	2.3 × 10^2^	0	0
Dong site	0	0	2.0 × 10^2^	0	0
Yu Dongquan site	0	0.2 × 10^2^	6.6 × 10	5 × 10^−1^	0

**Table 5:** Environmental factors measured at each sampling site.

Site	T (°C)	pH	A (m)	Dep (m)	H′_BM_	H′_P_	Tur	DO (mg/L)	F(m/s)
HH	21.87	8.36	802	0.98	1.257	1.3800	5.8	6.71	0.4122
Ban Fangzi	21.00	8.53	1074	0.41	2.273	0.6365	2.8	7.86	0.3744
Chang Ping	19.27	8.60	1141	0.05	2.312	1.1350	4.6	7.64	0.1674
Hou Zhenzi	16.70	8.43	1264	1.25	1.72	1.1630	3.9	7.46	0.1433
HT	20.31	8.53	1146	0.45	1.865	1.8360	5.7	7.78	0.4923
Chen Jiagou	17.28	8.46	645	0.23	1.382	0.6721	3.6	8.34	0.3053
Yu Dongquan	20.23	8.36	933	1.37	1.306	1.6420	4.7	7.22	0.1014
Hua Erping	20.76	8.78	1321	1.32	2.019	1.3860	3.9	6.65	0.1092
HD	18.98	8.34	1032	1.45	1.427	0.1914	3.7	7.49	0.1258

Note: T, Water temperature; A, Altitude of the sampling site; Dep, Water depth at the sampling point; H′_BM_, Shannon–Weiner index of Benthic Macroinvertebrates; H′_P_, Shannon–Weiner index of Phytoplankton; Tur, Turbidity; DO, Dissolved Oxygen; F, Flow velocity.

**Table 6:** Correlations between *B. lenok tsinlingensis* biomass and environmental factors.

	Biomass	T	pH	A	Dep	H′_BM_	H′_P_	Tur	DO	F
Biomass	1									
T	−0.1994	1								
pH	−0.2158	0.1768	1							
A	0.0186	0.0267	0.5814	1						
Dep	0.5348	0.0581	−0.2667	0.2493	1					
H′_BM_	−0.3309	0.1207	0.7480	0.6570	−0.4853	1				
H′_P_	−0.5447	0.3510	0.2288	0.2319	0.0354	0.0070	1			
Tur	−0.2812	0.3768	−0.1771	−0.1120	−0.0025	−0.3114	0.7356	1		
DO	−0.0429	−0.5539	−0.1443	−0.3254	−0.6646	0.1529	−0.4026	−0.3788	1	
F	−0.5009	0.3610	−0.0808	−0.3294	−0.5483	0.0350	0.1984	0.3759	0.2861	1

Note: T, Water temperature; A, Altitude of the sampling site; Dep, Water depth at the sampling point; H′_BM_, Shannon-Weiner index of Benthic Macroinvertebrates; H′_P_, Shannon-Weiner index of Phytoplankton; Tur, Turbidity; DO, Dissolved Oxygen; F, Flow velocity.

## Data Availability

The raw data supporting the conclusions of this article will be made available by the authors on request.

## Supplementary Materials

The following supporting information can be downloaded at: https://www.mdpi.com/article/10.3390/ani16131957/s1, Table S1: Phytoplankton abundance value; Table S2: Zooplankton abundance value; Table S3: Benthic macroinvertebrate abundance value; Table S4: Habitat photographs of the 14 sampling sites.
