# Supplementary material for: eDNA-qPCR Reveals Spatial Biomass and Habitat Associations of the Endangered Brachymystax lenok tsinlingensis in Zhouzhi Heihe River
Source: Animals (Basel). 2026 Jun 24;16(13):1957. doi: 10.3390/ani16131957 (PMC13360022; doi:10.3390/ani16131957)
Supplement: Supplementary file 1 [file animals-16-01957-s001.zip › Table S4 Habitat photographs of the 14 sampling sites.pdf]

Table S4 Habitat photographs of the 14 sampling sites

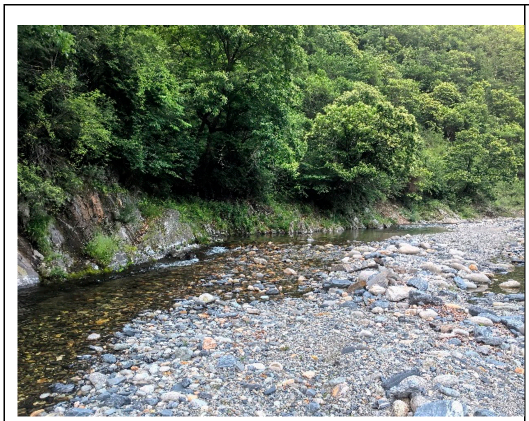	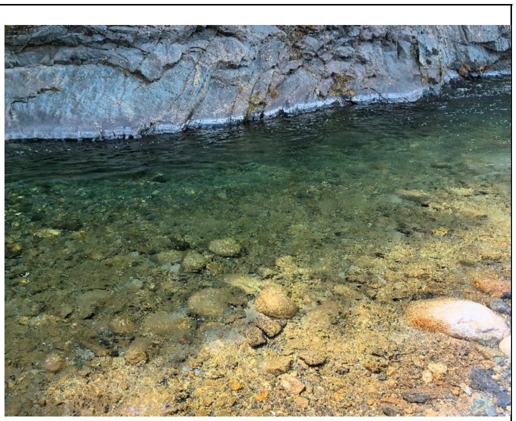
**Hua Erping site**	**Hou Zhenzi site**
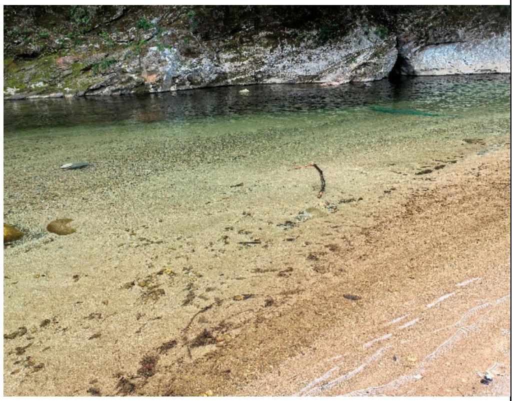	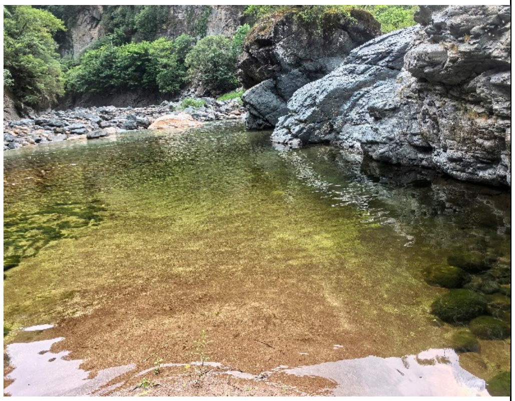
**HT site**	**HD site**
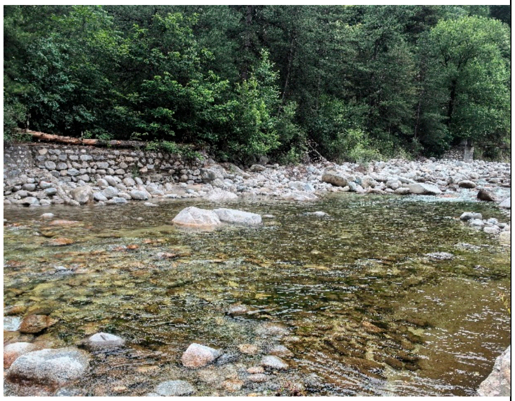	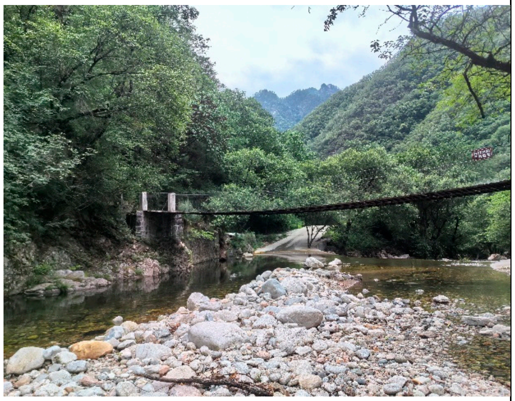
**Da Manggou site**	**Ba Muping site**

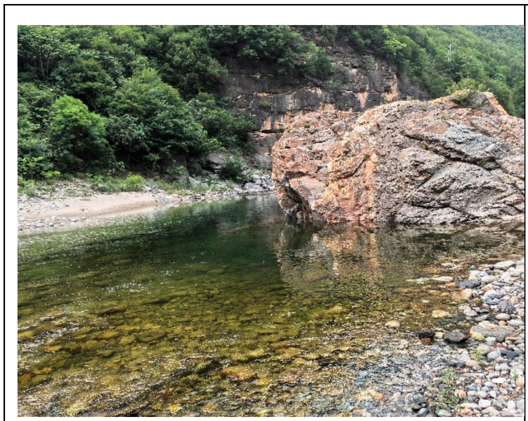

Yu Dongquan site

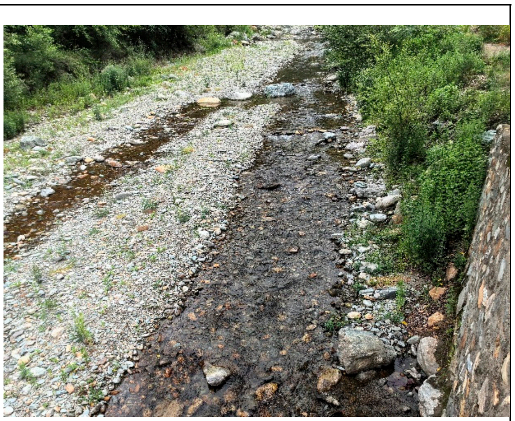

Chang Ping site

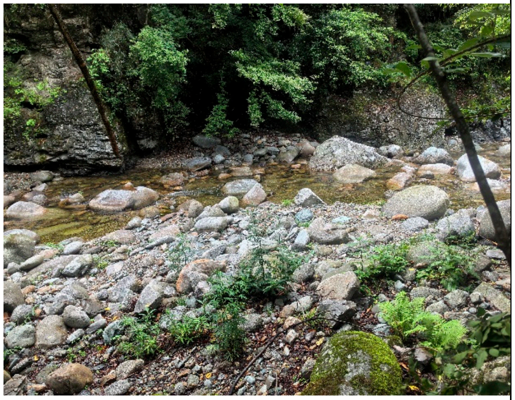

Bang Fangzi site

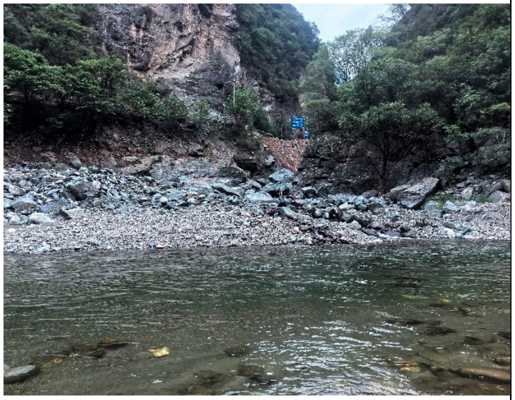

HH site

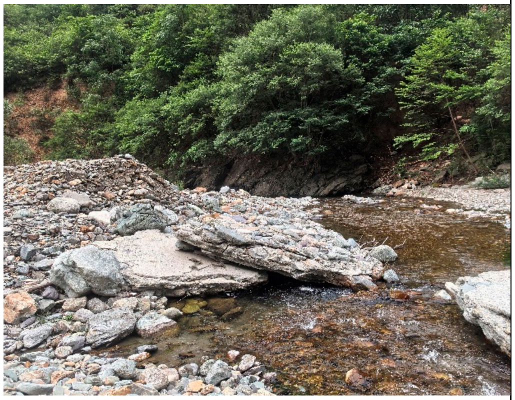

Hu Bao site

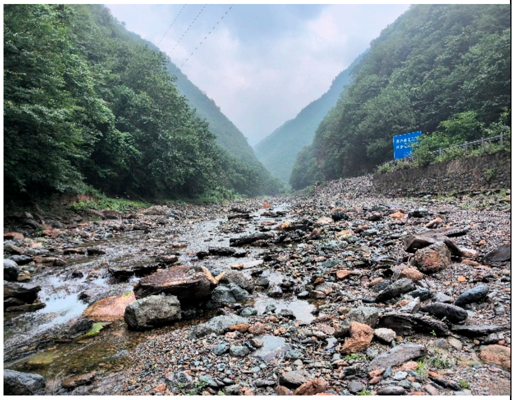

Wang Jia site

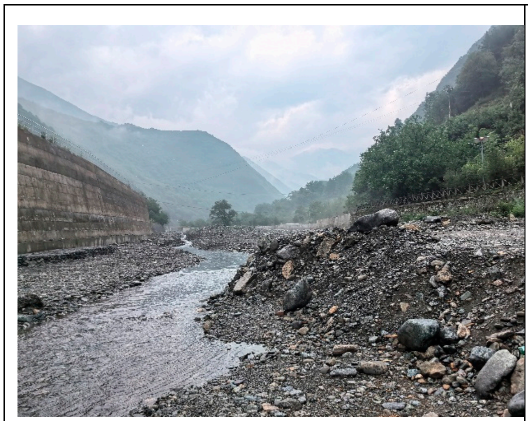	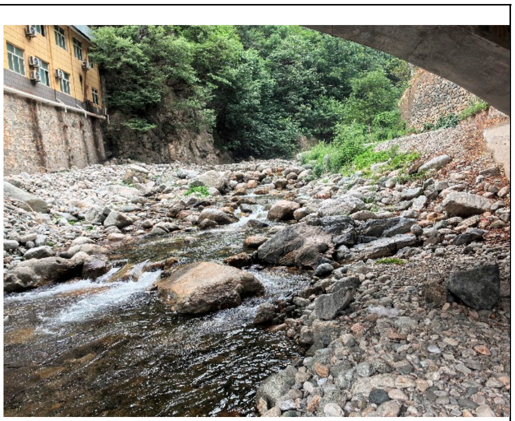
**Dong site**	**Chen Jiagou site**
